# Arithmetic optimization algorithm based maximum power point tracking for grid-connected photovoltaic system

**DOI:** 10.1038/s41598-023-32793-0

**Published:** 2023-04-12

**Authors:** Mohamed Ahmed Ebrahim Mohamed, Shymaa Nasser Ahmed, Mohamed Eladly Metwally

**Affiliations:** 1 Department of Electrical Engineering, Faculty of Engineering at Shoubra, Benha University, Cairo, Egypt; 2 Department of Electrical Engineering, El Shorouk High Institute of Engineering, Cairo, Egypt

**Keywords:** Energy science and technology, Engineering

## Abstract

This paper suggests an optimal maximum power point tracking (MPPT) control scheme for a grid-connected photovoltaic (PV) system using the arithmetic optimization algorithm (AOA). The parameters of the proportional-integral (PI) controller-based incremental conductance (IC) MPPT are optimally selected using AOA. To accomplish this study, a 100-kW benchmark PV system connected to a medium distribution utility is constructed and analyzed employing MATLAB/SIMULINK. The optimization framework seeks to minimize four standard benchmark performance indices, then select the best of the best among them. To verify the efficacy of the recommended methodology, a comprehensive comparison is conducted between AOA-based PI-IC-MPPT, modified incremental conductance MPPT (MIC), grey wolf optimization (GWO), genetic algorithm (GA), and particle swarm optimization (PSO)-based MPPT. The proposed control approach has achieved a reduction of 61, 3, 4.5, and 26.9% in the rise time and a decrease of 94, 84.7, 86.6, and 79.3% in the settling time compared with MIC, GWO, GA, and PSO in extracting MPPT of the proposed system, respectively.

## Introduction

There is an urgent destination toward dependency on renewable energy resources in power generation worldwide because they don’t pollute the environment with Co_2_ emissions and are abundantly available in contrast to fossil fuels^1^. The total cumulative renewable energy production capacity was 3064 GW in 2021^2^. The extra renewable supplying power reached 257 GW in 2021^2^. One of the greatest significant resources of green energy is photovoltaic (PV)^3^. The PV occupies the first rank in 2021 with a contribution of 133 GW from 257 GW of additional capacity, as declared in the international renewable energy agency (IRENA) statistics^2^.

However, the major challenge with PV systems is dealing with the observed nonlinear properties of current–voltage (I-V) and power voltage (P–V) relationships. Besides, their output power is essentially influenced by dominant variations of climate weather, such as temperature and irradiance^4^. So, it is crucial to track the peak PV output related to solar irradiance and surrounding temperature by implementing different maximum power point tracking (MPPT) schemes^5–7^. The MPPT methods are categorized as conventional, artificial intelligence (AI), optimization, or hybrid MPPT^8^. The most prevalent methods of classical MPPT are incremental conductance (IC), fractional short circuit current (FSCC), fractional open-circuit voltage (FOCV), and perturb and observe (P&O)^9–12^. The AI-based MPPT techniques include artificial neural networks and fuzzy logic^8^. The optimization-based MPPT schemes are such as Harris hawk optimization^1^, Improved Grey Wolf Optimizer^13^, and improved squirrel search algorithm^14^. The hybrid MPPT techniques that merge conventional and AI-based MPPT techniques to combine the merits of each are presented in^15,16^ or combine two or more AI or optimization algorithms as in^17–20^.

These various MPPT procedures differ in terms of affordability, convergence rate, requisite sensors, complication rate, and popularity^21^. P&O and IC-based MPPT schemes are widely implemented because of their simplicity and affordability^22–24^. However, P&O produces oscillations around MPP and wrong decisions in case of sudden insolation change^25^, while the IC-based MPPT methods had superior performance over P&O in extracting MPP during sudden atmospheric conditions^26^. Fixed-step IC has drawbacks: if the step is small, it reduces the speed of convergence, while if the size of the step is large, it generates large losses and oscillations^27^. These shortcomings can be solved by implementing the variable step size method; the step size decision is based on the operating point; if the operating point is far from the MPP, the step size will be large, while the step is small when the operating point is close to the MPP to balance between oscillations around MPP and speed of convergence^28–30^.

To extract the maximum output of the PV system, the PV array voltage must be adjusted to its pre-calibrated maximum power point voltage^31^. The PV array voltage is adjusted by modifying the DC-DC converter duty ratio that has been accomplished through a control technique such as proportional-integral (PI)^32^, proportional-derivative (PD), proportional-integral-derivative (PID)^33^, fuzzy logic^34^, and slide mode controllers^35^. Fuzzy and neural controllers provide an efficient MPPT performance but fuzzy is controlled by linguistic rules that incorporate professional expertise and knowledge, such as fuzzy set definition, membership function shape selection, and rule table construction, all of which require better expertise and intuition from designers and have a direct impact on tracking speed and accuracy^11^. Moreover, the neural controllers required high data training for each PV array^36^.

Hence, PID controllers remain the most commonly used regulators in industrial applications with various structures such as PI or PD^37^. However, it is necessary to select the optimal gains of the PI controller properly^38^. In the last few years, various optimization techniques were employed to obtain gains of the PI controller^39^, including the Marine Predators Algorithm^40^, Particle Swarm Optimization^41^, Grey Wolf Optimization^42^, Henry Gas Solubility Optimization^43^, Grasshopper Optimization Algorithm^44^, Ant Lion Optimizer^45^, Genetic Algorithm^46^, and Enhanced Artificial Bee Colony Algorithm^47^ in different applications.

Table 1 clarifies the superiority of PI-based IC MPPT against various MPPT techniques. Hence, the paper proposed a new arithmetic optimization algorithm (AOA) for calculating PI-based IC MPPT of a 100 kW PV model linked to the grid.

**Table 1:** Comparison of Various MPPT Methods against IC with a PI Controller.

Authors	MPPT strategy	DC/DC converter	Application	Compared with	Advantages of IC with PI controller over other techniques
^48^	IC with PI controller	Boost converter	On-grid	P&O, FSCC, and FOCV	IC achieved the maximum available power at different climate weather scenarios compared with all other techniques
					IC had a faster tracking response compared with FSCC and FOCV
^49^		Boost converter	On-grid	P&O, and classical IC	IC attained maximum efficiency
					IC had minimum settling time consumption
					IC had the best steady state and transient response
^50^		Zeta converter	Stand-alone	classical IC	IC had less tracking time
					IC response was high accuracy with negligible oscillation, while the classical one (large step has very noticeable oscillation and small step has longer tracked time)

The major benefit of such implemented optimization is that it opens up a wide search space^51^. The four standard performance indices, including integral time absolute error (ITAE), integral absolute error (IAE), integral time square error (ITSE), and integral square error (ISE), have been employed in this paper with selecting the best of the best of them. The primary accomplishments of this paper are as follows:

1. Up to the knowledge of the authors, this is the first time to rely on the AOA for the optimal design of PI-IC-MPPT for a grid-connected PV system.
2. The authors consider four benchmark standard indicators (IAE, ISE, ITAE, and ITSE) to select the best of each index and then select the best of the best index among them.
3. The suggested control scheme is evaluated and validated through a 100-kW benchmark PV system linked to the grid.
4. The developed AOA-based PI-IC-MPPT control scheme is tested and validated under small, large, and realistic weather conditions.
5. A comparison is carried out among the findings of the proposed control strategy, grey wolf optimization (GWO), modified incremental conductance (MIC), genetic algorithm (GA), and particle swarm optimization (PSO) to prove the effectiveness of the adopted control scheme.

The remaining of the article is arranged as follows: PV modeling and system configuration are clarified in Sections “Modeling of photovoltaic” and “System configuration”. Further, In Sections “Incremental conductance MPPT” and “PI controller”, the IC MPPT algorithm and PI-based IC MPPT are explained. Besides, In Section “Method”, the arithmetic optimization algorithm and grey wolf optimization are illustrated. In Section “Simulation framework”, the simulation framework of the case under study is presented. Moreover, Section “Results and Discussion”, presents and analyses the simulation results. Lastly, Section “Conclusion”, offers the conclusion.

## Modeling of photovoltaic

**Figure 1:**
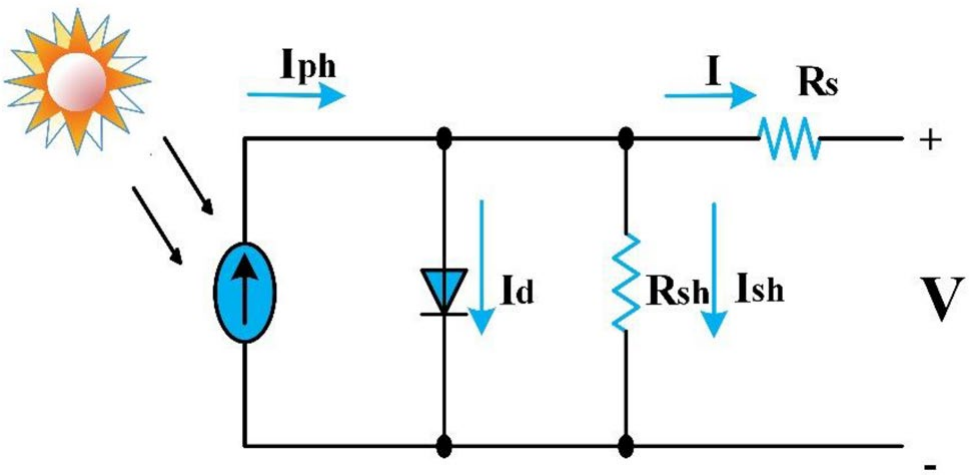
Single Diode PV Cell Model.

Researchers devised various schemes for modeling the PV cell, involving single, double, and triple diode models (SDM, DDM, and TDM)^52^. But the simplest widespread model is SDM due to it requires minimal estimation of the equivalent circuit parameters^53^. Figure 1 depicts the SDM PV cell.

The mathematical equations that represent the PV module that consist of series-connected PV cells in SDM can be expressed by Eqs. (1, 2, 3, 4, 5 and 6)^54^.

1 $${\varvec{I}}={{\varvec{I}}}_{{\varvec{p}}{\varvec{h}}}-{{\varvec{I}}}_{{\varvec{d}}}-{{\varvec{I}}}_{{\varvec{s}}{\varvec{h}}}$$

2 $${\varvec{I}}={{\varvec{I}}}_{{\varvec{p}}{\varvec{h}}}-{{\varvec{I}}}_{{\varvec{o}}}\left(\mathbf{exp}\left(\frac{{\varvec{V}}+{{\varvec{R}}}_{{\varvec{s}}}{\varvec{I}}}{{\varvec{a}} {{\varvec{N}}}_{{\varvec{s}}}{ {\varvec{V}}}_{{\varvec{t}}}}\right)-1\right)-\frac{{\varvec{V}}+{{\varvec{R}}}_{{\varvec{s}}} {\varvec{I}}}{{{\varvec{R}}}_{{\varvec{s}}{\varvec{h}}}}$$

3 $${{\varvec{V}}}_{{\varvec{t}}}= \frac{{\varvec{K}} {{\varvec{T}}}_{{\varvec{c}}}}{{\varvec{q}}}$$

4 $${{\varvec{I}}}_{{\varvec{p}}{\varvec{h}}}= \frac{{\varvec{G}}}{{{\varvec{G}}}_{{\varvec{n}}}}\left({{\varvec{I}}}_{{{\varvec{s}}{\varvec{c}}}_{{\varvec{n}}}}+{{\varvec{K}}}_{{\varvec{T}}}\left({{\varvec{T}}}_{{\varvec{c}}}-{{\varvec{T}}}_{{{\varvec{c}}}_{{\varvec{n}}}}\right)\right)$$

5 $${{\varvec{I}}}_{{\varvec{o}}}= {{\varvec{I}}}_{{{\varvec{o}}}_{{\varvec{n}}}}\left(\frac{{{\varvec{T}}}_{{\varvec{c}}}}{{{\varvec{T}}}_{{{\varvec{c}}}_{{\varvec{n}}}}}\right)^{3} {\varvec{e}}{\varvec{x}}{\varvec{p}}\left(\frac{{\varvec{q}}{{\varvec{E}}}_{{\varvec{g}}}}{{\varvec{a}}{\varvec{k}}}\left(\frac{1}{{{\varvec{T}}}_{{{\varvec{c}}}_{{\varvec{n}}}}}-\frac{1}{{{\varvec{T}}}_{{\varvec{c}}}}\right)\right)$$

6 $${{\varvec{I}}}_{{{\varvec{o}}}_{{\varvec{n}}}= }\frac{{{\varvec{I}}}_{{{\varvec{s}}{\varvec{c}}}_{{\varvec{n}}}}}{{\varvec{e}}{\varvec{x}}{\varvec{p}}\left(\frac{{{\varvec{V}}}_{{{\varvec{o}}{\varvec{c}}}_{{\varvec{n}}}}}{{\varvec{a}}{{\varvec{N}}}_{{\varvec{s}}}{ {\varvec{V}}}_{{{\varvec{t}}}_{{\varvec{n}}}}}\right)}$$

where ${I}_{ph}$ is photogenerated current, ${I}_{d}$ is Shockley diode current, ${I}_{sh}$ is current through parallel resistance, ${I}_{o}$ is saturation current of diode, $V$ is terminal voltage, ${R}_{s}$ is series resistance, $a$ is the ideality factor for diode = 0.94504, ${N}_{s}$ is the number of series cells, ${V}_{t}$ is the thermal voltage, ${R}_{sh}$ is shunt resistance, $K$ is Boltzmann constant = 1.38 × 10^−23^ J/K, ${T}_{c}$ is temperature of PV cell in Kelvin, $q$ is electron charge = 1.6 × 10^−19^ C, $G$ is solar irradiance, ${I}_{{sc}_{n}}$ is a short circuit current at standard test condition(STC) that symbolized by $\left(n\right)$ which means (${G}_{n}$ = 1000 w/m^2^, ${T}_{{c}_{n}}$ = 25 °C), ${K}_{T}$ is the temperature coefficient of ${I}_{sc}$, ${E}_{g}$ is bandgap energy of polycrystalline silicon = 1.12 eV at 25 °C, and ${V}_{{oc}_{n}}$ is open circuit voltage.

## System configuration

**Figure 2:**
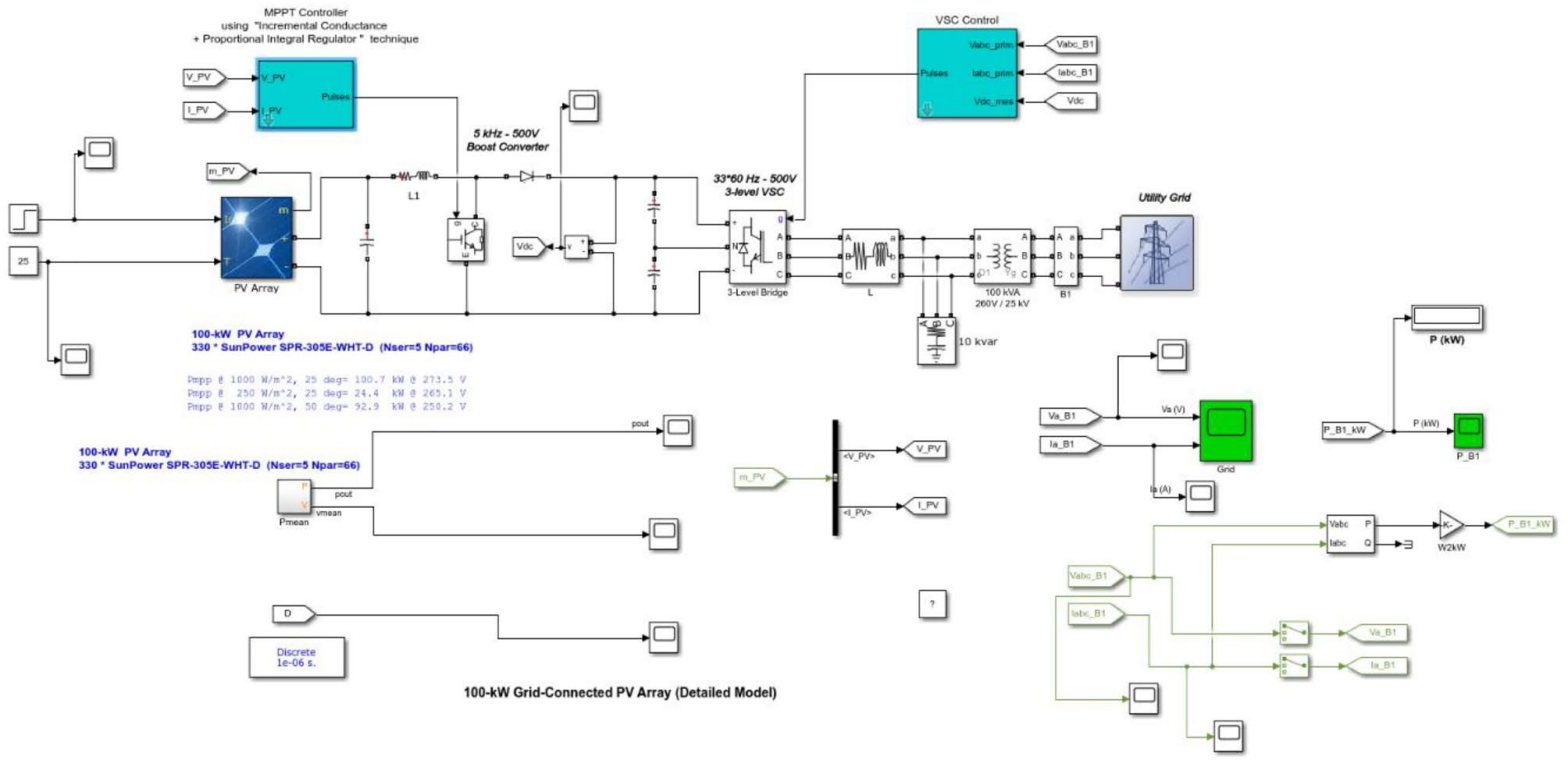
System Construction.

**Figure 3:**
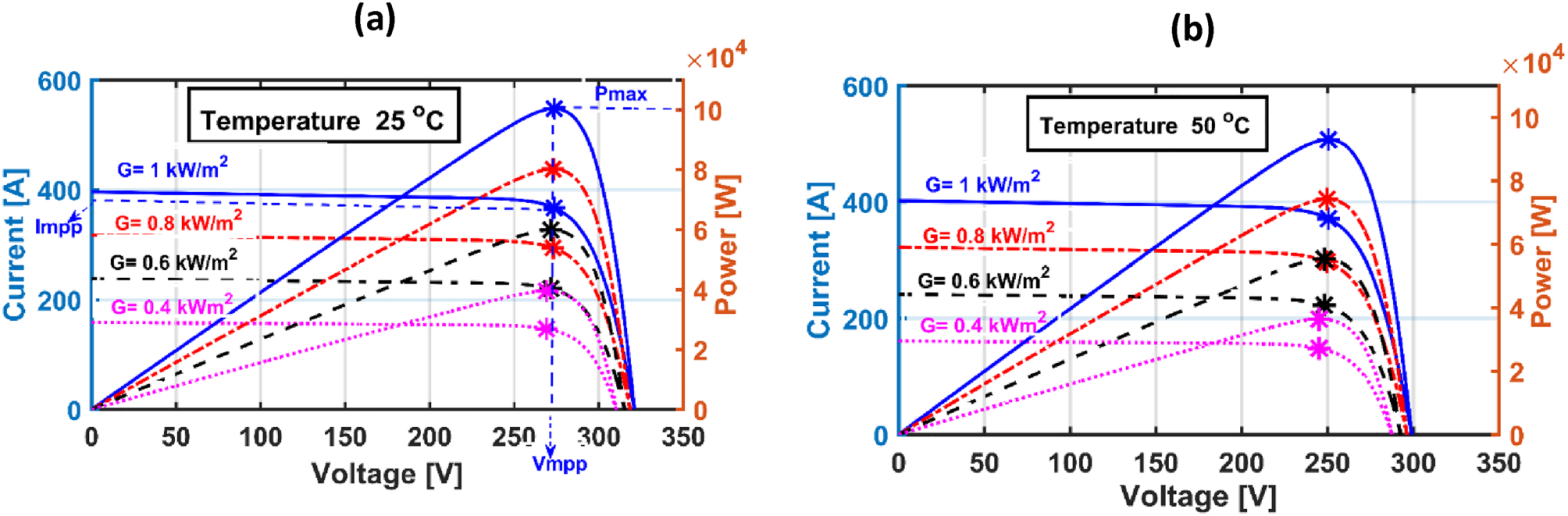
I-V and P–V characteristic curves for a 100 kW PV system (**a**) at 25 °C with multiple irradiances, (**b**) at similar previous irradiances with a specified temperature of 50 °C.

The capacity of the PV system under study is 100 kW. The PV array comprises five shunt-connected strings; each string contains sixty-six series-connected modules of type SunPower SPR-305E-WHT-D. The proposed PI-based IC MPPT regulator tuned by AOA is applied to minimize the error signal of the conductance for producing the maximum output of the PV. Therefore, the PI regulator corrects the duty cycle of the 500 V boost converter that is tied to the inverter linked to the medium grid. Figure 2 displays a block schematic for the suggested approach. Besides, I–V and P–V relationships concerning various climate weather scenarios are displayed in Fig. 3. It is noticeable that PV output relies mostly on temperature and irradiance^55^.

## Incremental conductance MPPT

**Figure 4:**
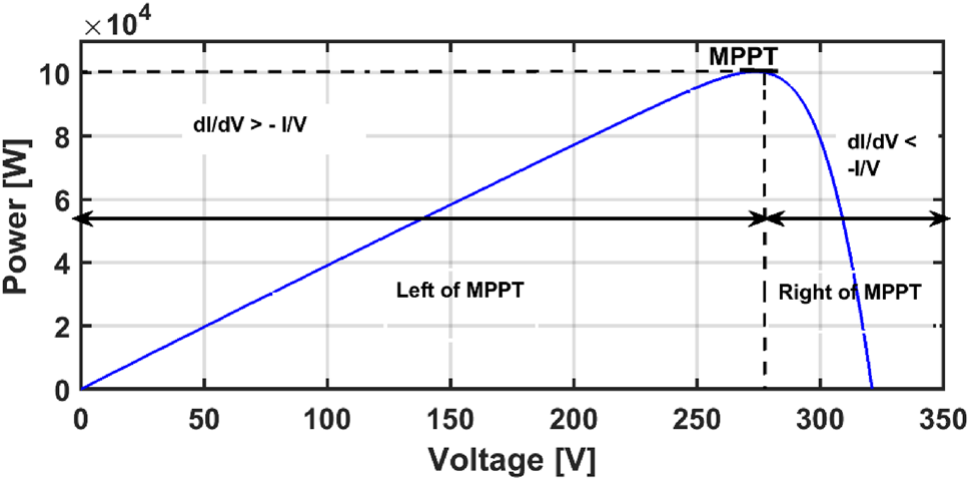
Principle of IC MPPT.

The IC is one of the most popular classical MPPT techniques that is based on contrasting the momentary conductance (I/V) with the incremental conductance(dI/dV)^56^. The mathematical equations that represent IC can be summarized in Eq. (7), where if (dI/dV) is equal to -I/V, then the PV array operates at the MPP; if dI/dV is greater than -I/V, the PV array is located at the left of the MPP; and if the dI/dV is less than -I/V, the PV array operates at the right side of the MPP, as presented in Fig. 4.

7 $$\left\{\begin{array}{l}dI/dV=-I/V\, at\, MPP \quad \,slope\,=\,0\\ dI/dV>-I/V\, at\, left\, of\, MPP\, \quad slope\,=\,+ve\\ dI/dV<-I/V\, at\, right\, of\, MPP\, \quad slope\,=\,-ve\end{array}\right.$$

## PI controller

PI controllers have been extensively deployed in different industrial fields because of their simplicity, ease of implementation, and robust performance^37^. A finely tuned PI controller means getting optimum values of the two gains. The first factor is proportional gain (K_P_), while the second is integral gain (K_I_). Researchers utilize many optimization algorithms to tune PI controllers, such as the whale optimization algorithm, genetic, cuckoo search, and Artificial Bee Colony due to eliminate the error associated with PV MPPT techniques^57–59^.

The optimization cost function minimizes the error signal $e(t)$ produced by IC to guarantee the best MPPT performance using the four standard indicators IAE, ISE, ITAE, and ITSE, using the expression Eq. (8) to more accurately convey the superior results of the suggested control technique^60^.

8 $$\left\{\begin{array}{l}IAE = {\int }_{0}^{{t}_{ss}}|e(t)| .dt\\ ISE = {\int }_{0}^{{t}_{ss}}{e}^{2}(t) .dt\\ \genfrac{}{}{0pt}{}{ ITAE = {\int }_{0}^{{t}_{ss}}t.|e(t)| .dt}{ ITSE = {\int }_{0}^{{t}_{ss}}{t.e}^{2}(t) .dt}\end{array}\right.$$

where ${t}_{ss}$ is the steady state time response and $e(t)$= $dI(t)/dV(t)+I(t)/V(t)$.

## Method

### Arithmetic optimization algorithm

**Figure 5:**
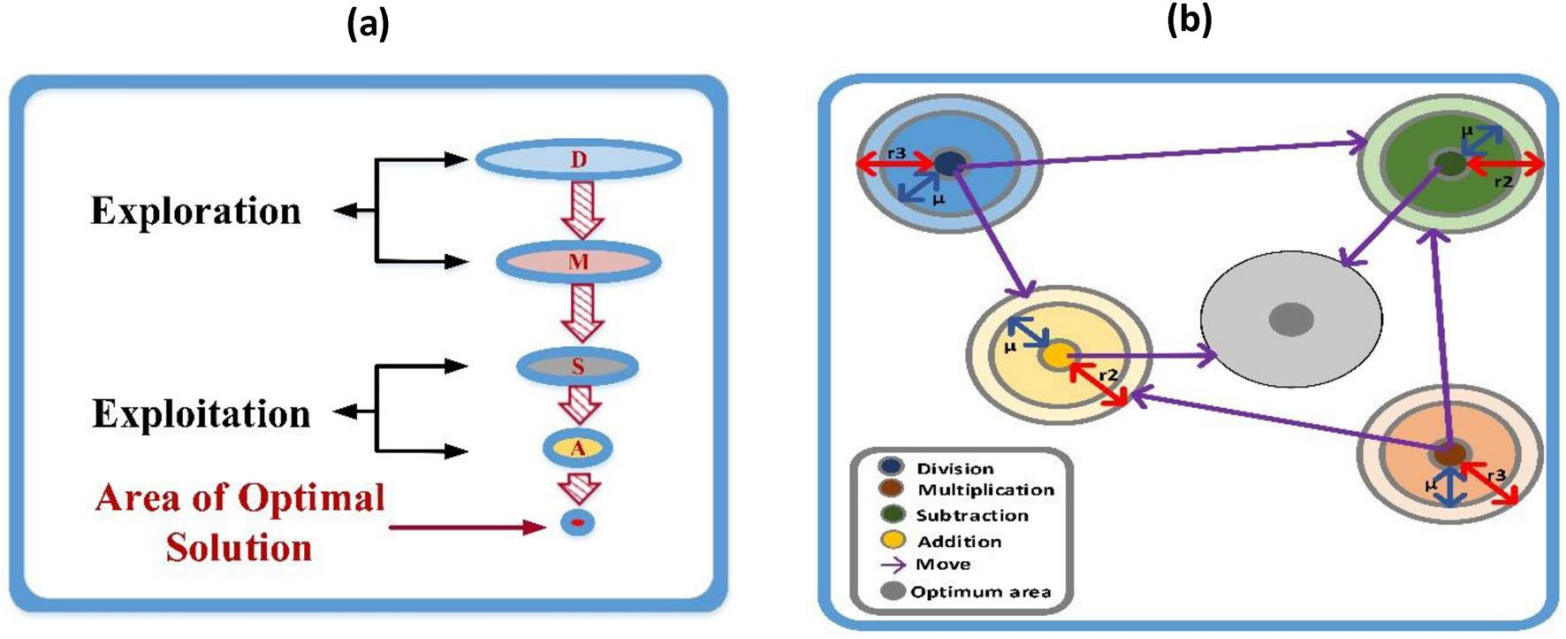
(**a**) Hierarchy of arithmetic operators, (**b**) Model for changing arithmetic operators' positions in AOA toward the optimal area.

Abualigah suggests a novel meta-heuristic optimization termed “AOA” in 2021^61^. The use of mathematical operators in solving math problems served as the inspiration for the AOA. These basic arithmetic operators include addition (A), multiplication (M), subtraction (S), and division (D). Such an AOA involves two steps: the first is exploration, followed by exploitation, as shown in Fig. 5a.

### Initialization

The AOA optimization method initiates with a matrix involving random nominated solutions ($X$), as written in Eq. (9). The finest one at each repetition is regarded as the optimum solution so far.

9 $$X=\left[\begin{array}{ccc}{x}_{\mathrm{1,1}}& \cdots & {x}_{1,n}\\ \vdots & \ddots & \vdots \\ {x}_{N,1}& \cdots & {x}_{N,n}\end{array}\right]$$

Before the beginning of AOA algorithm, search stages such as intensification and diversification must be selected. Hence, the Math Optimizer Accelerated function (MOA) is computed during the next searching stages according to Eq. (10).

10 $$MOA({C}_{\_}Iter)=Min+{C}_{\_}Iter\frac{Max-Min}{M\_Iter}$$

The terms $Min$ and $Max$ represent the lowest and extreme values of the accelerated function, correspondingly. $MOA(\mathrm{C}\_Iter)$ indicates the value of the function at recent repetition. $C\_Iter$ denotes the current repetition range between one and the maximum number of iterations ($M\_Iter$).

### Exploration phase

The exploration phase is accomplished by (M) or (D) arithmetic operators as they have widely distributed values (referring to various fields). However, as a cause of the wide dispersion of such operators (M and D), these operators cannot easily close the target dissimilar to other operators such as (S and A). To illustrate the influence of the various operators’ distribution values, a function is established by four arithmetic processes. Consequently, the exploration step benefits the identification of a close-optimum solution, which can be found after multiple iterations. Besides, such a process aids the exploitation step in the search procedure through improved communication. This phase is executed if $r1$> $MOA$, where r1 is a value selected randomly from the range [0,1]. The updated location is applied by the D operator if $r2$ < 0.5 ($r2$ is another number chosen randomly from the range [0,1]), and the other M operator is negligible till this operator ends its mission. Otherwise, the position is updated using the M operator. The arithmetic representation of this search phase is expressed according to Eq. (11).

11 $${x}_{i,j}(C\_Iter +1)=\left\{\begin{array}{l}best\left({x}_{j}\right)\div \left(MOP+ \epsilon \right) \\ \times \left(\left({UB}_{j}-{LB}_{j}\right)\times \mu +{LB}_{j} \right), \quad r2<0.5 \\ best\left(xj\right)\times \left(MOP+ \epsilon \right) \\ \times \left(\left(UBj-LBj\right)\times \mu +{LB}_{j}\right), \quad otherwise\end{array}\right.$$

where ${x}_{i,j}(C\_Iter +1)$ is the solution of the subsequent repetition, $best\left({x}_{j}\right)$ indicates the place of the optimal-attained solution till now, $\epsilon$ is a tiny number, ${UB}_{j} and {LB}_{j}$ are the top and the lower limits of the ${j}^{\mathrm{th}}$ location correspondingly. $\mu$ is a controlling factor purposed to modify the search process, which is set to 0.499 based on the tests, MOP represents the math optimizer probability coefficient that is calculated from Eq. (12).

12 $$MOP\left(C\_Iter\right)=1-{(C\_\_Iter)}^{1/\propto }{/(M\_Iter)}^{1/\propto }$$

where $\alpha$ is a delicate factor that determines the effectiveness of the exploitation throughout the iterations, and it is designed at 5 after many attempts.

### Exploitation phase

The exploitation phase is performed by (S) or (A) mathematical operators as they have highly concentrated results. The condition for this phase is $r1$< $MOA$. Operator S is responsible for updating the position if r3 < 0.5 ($r3$ is a random value between [0,1]) and the other (A) disregarded till this operator completes its target. Else, the position is updated by the (A) operator. The mathematical model of this search phase is represented according to Eq. (13). To keep the exploration running throughout the initial and last trials, the parameter $\mu$ is precisely designed to provide a randomized result at each repetition. Figure 5b illustrates the manner of updating the location of a search solution based on D, M, S, and A operators.

13 $${x}_{i,j}(C\_Iter +1)=\left\{\begin{array}{l}best\left({x}_{j}\right)-\left(MOP+ \epsilon \right) \\ \times \left(\left({UB}_{j}-{LB}_{j}\right)\times \mu +{LB}_{j} \right), \quad r3<0.5 \\ best\left(xj\right)+\left(MOP+ \epsilon \right) \\ \times \left(\left(UBj-LBj\right)\times \mu +{LB}_{j}\right), \quad otherwise\end{array}\right.$$

The steps of AOA can be summarized as:

Step 1: Select the suitable population, design parameters of AOA (α = 5, $\mu$ =0.499), and define the maximum permitted iterations.

Step 2: Set initially the positions of solutions at random.

Step 3: Calculate the objective function for such solutions as in Eq. (8), then select the best of them and set it as the best solution thus far.

Step 4: Update $MOA$ and $MOP$ as in Eqs. (10, 12), respectively.

Step 5: Create three random numbers ($r1$, $r2$, and $r3$).

Step 6: Update the position of solutions by D operator if $r1$> $MOA$ and $r2$<0.5 using Eq. (11) or adjust the location of solutions by the M operator if $r1$> $MOA$ and $r2$>0.5 using Eq. (11) or adjust the location of solutions by the S operator if $r1$< $MOA$ and $r3$<0.5 using Eq. (13) or adjust the location of solutions by the A operator if $r1$< $MOA$ and $r3$>0.5 using Eq. (13).

Step 7: Calculate the new objective function for the updated position of solutions and exchange them if the novel solutions are fitter than the previous.

Step 8: Display the optimal solution (K_P_, K_I_) if the recent iteration is equated to the limit constraint.

The main advantages of the applicable AOA over the existing optimization techniques can be summarized as follows: (i) It is a new optimization algorithm with a basic structure, simply including a few mathematical operations, and there are just two control factors necessary^62^. (ii) It has a wide search space in the exploration phase. On the other hand, some updates and modifications are needed for AOA to enhance its performance during the exploitation phase. Moreover, AOA likes any optimization technique that has no unique solution for any optimization problem. According to the no free lunch theory, there is no specific optimization method capable of solving all optimization problems, which means optimization results are case-dependent.

### Grey wolf optimization

**Figure 6:**
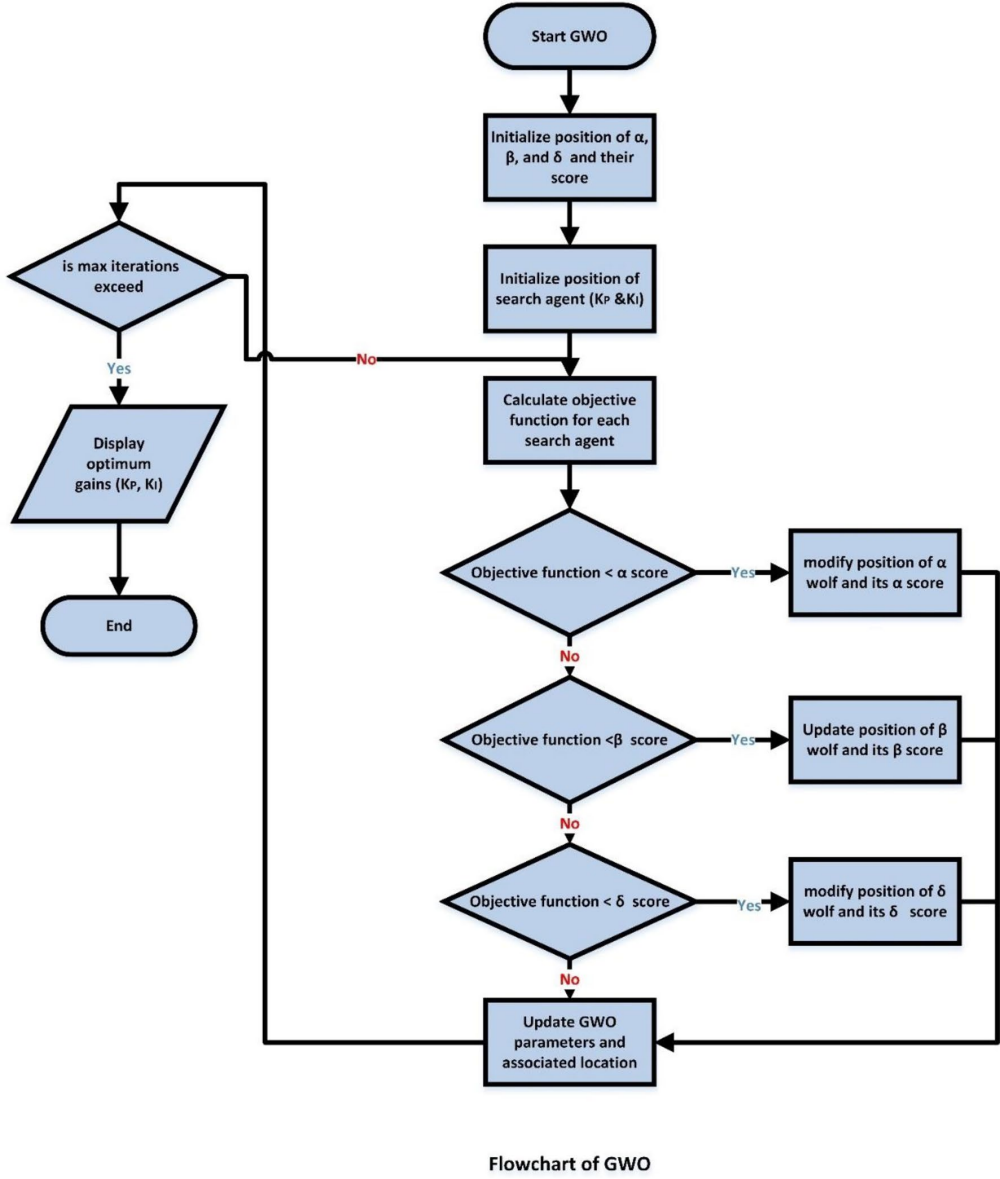
Flowchart of GWO.

GWO is an optimal procedure that Mirjalili et al. suggested in 2014^63^. This optimization algorithm draws inspiration from the behavior of grey wolves. Such GWO simulates the style of prey catching by grey wolves as well as the leadership structure. The leadership hierarchy of such animals is composed of four categories: alpha wolf (α) occupies the top rank, followed by the beta wolf (β), then delta (δ), and omega wolf (ω) exists in the lowest rank. The flowchart that describes the GWO procedure is depicted in Fig. 6.

### Parameters setting for optimization process

The maximum number of iterations ($M\_Iter$) and the search agent of each algorithm are selected after several attempts for suitable performance. In general, as $M\_Iter$ and the search agent increased, the accuracy of the obtained results increased, while the time also increased. The lower and upper boundaries were selected after many tries, starting with a wide boundary and changing it if the results were not the best until reaching the suitable boundary. For fair judgment, the same number of iterations, search agent, and lower and upper boundaries are selected for each algorithm as demonstrated in Table 2.

**Table 2:** Parameters of optimization algorithms.

Optimization technique	Parameters	Design	Number of iterations	Number of search agent	Lower and upper variables bound
GWO	$a$	$2-\frac{2}{M\_Iter}$	200	20	0 $<{\mathrm{K}}_{P}<$ 0.1 0.8 $<{\mathrm{K}}_{I}<$ 2
PSO	${C}_{1}$	2	200	20	
	${C}_{2}$	2			
	${C}_{0}$	0.65			
AOA	$\alpha$	5	200	20	
	$\mu$	0.499			
	$MOP(max)$	1			
	$MOP(min)$	0.2			

### Simulation framework

The simulation framework description can be explained in the following steps:

Step 1: Select the appropriate search agent and iterations restricted for AOA, GWO, GA, and PSO.

Step 2: Select the proper upper and lower boundaries of PI controller gains (K_P_, K_I_) for accurate and fast performance.

Step 3: Set $IAE$, $ISE$ $,ITAE$ $, \,and\, ITSE$ as a cost function extracted from the Simulink.

Step 4: Run AOA, GWO, GA, and PSO on the PV simulation model subjected to mentioned limitations above.

Step 5: Substitute with the optimum obtained parameters in the simulation model.

Step 6: Select the best gains.

**Figure 7:**
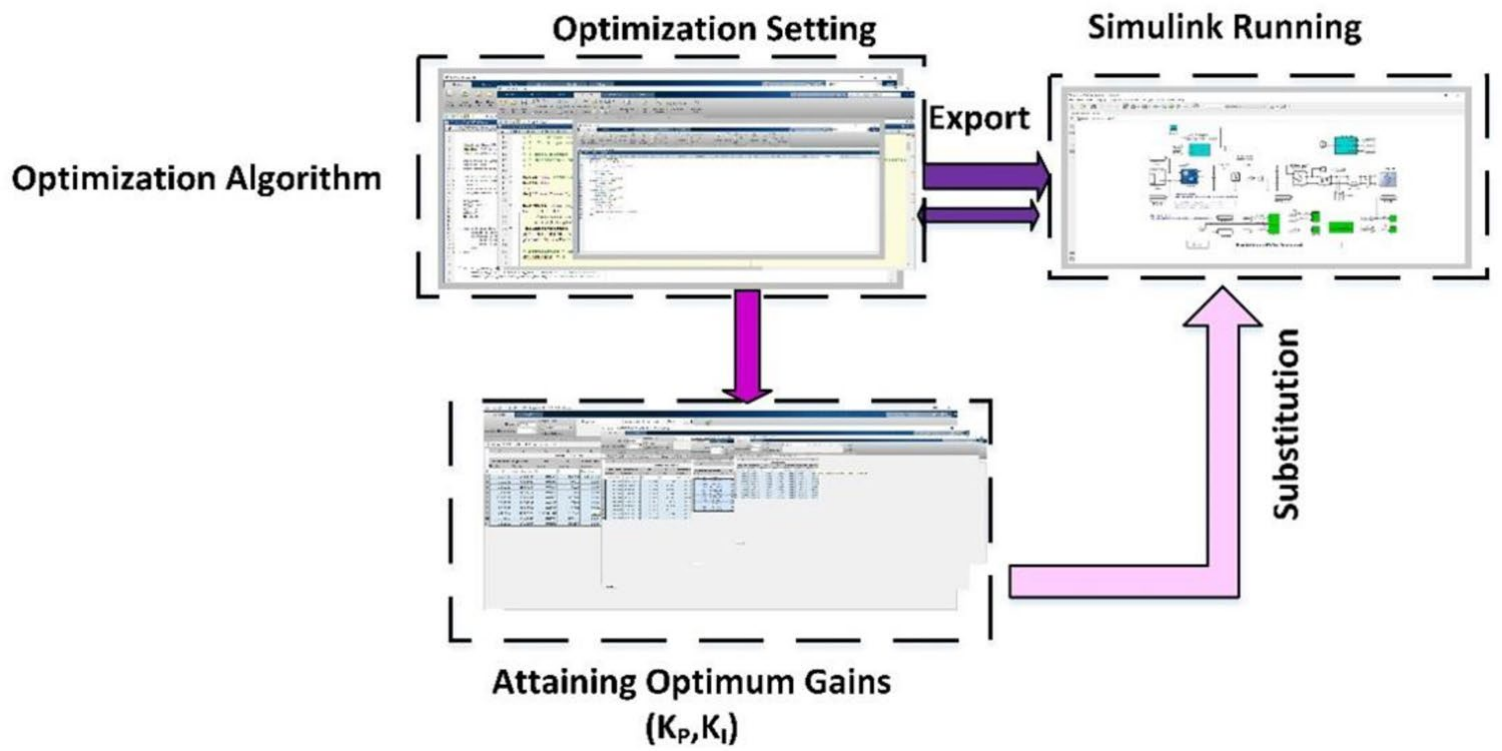
Simulation framework.

The simulation framework of the proposed system is depicted in Fig. 7.

### Simulation environment

The details of the simulation environment are provided in Table 3.

**Table 3:** Simulation environment details.

Software	MATLAB R2018a
Sampling control size	10^–4^ s
Solver type	Type (fixed step (auto)) Solver (discrete)
Powergui	Discrete with a sample time of 10^−6^ s
Processor	Intel(R) Core (TM) i7

## Results and discussion

**Figure 8:**
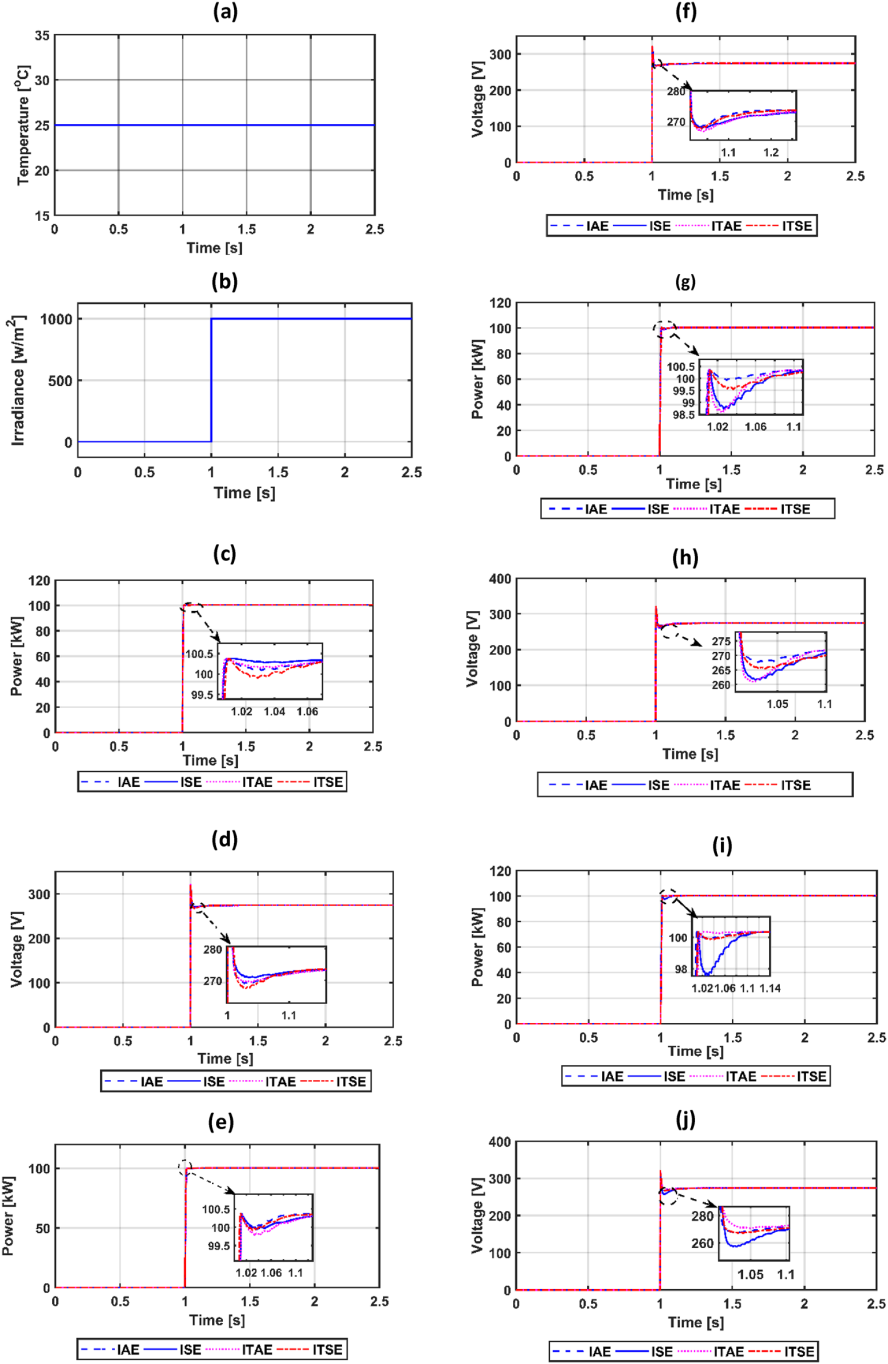
(**a**) constant temperature at 25 °C, (**b**) step irradiance, (**c**) PV output power-based AOA, (**d**) PV voltage-based AOA, (**e**) PV power-based GWO, (**f**) voltage of the PV-based GWO, (**g**) PV power -based GA, (**h**) voltage of the PV-based GA, (**i**) PV power-based PSO, (**j**) voltage of the PV-based PSO.

**Figure 9:**
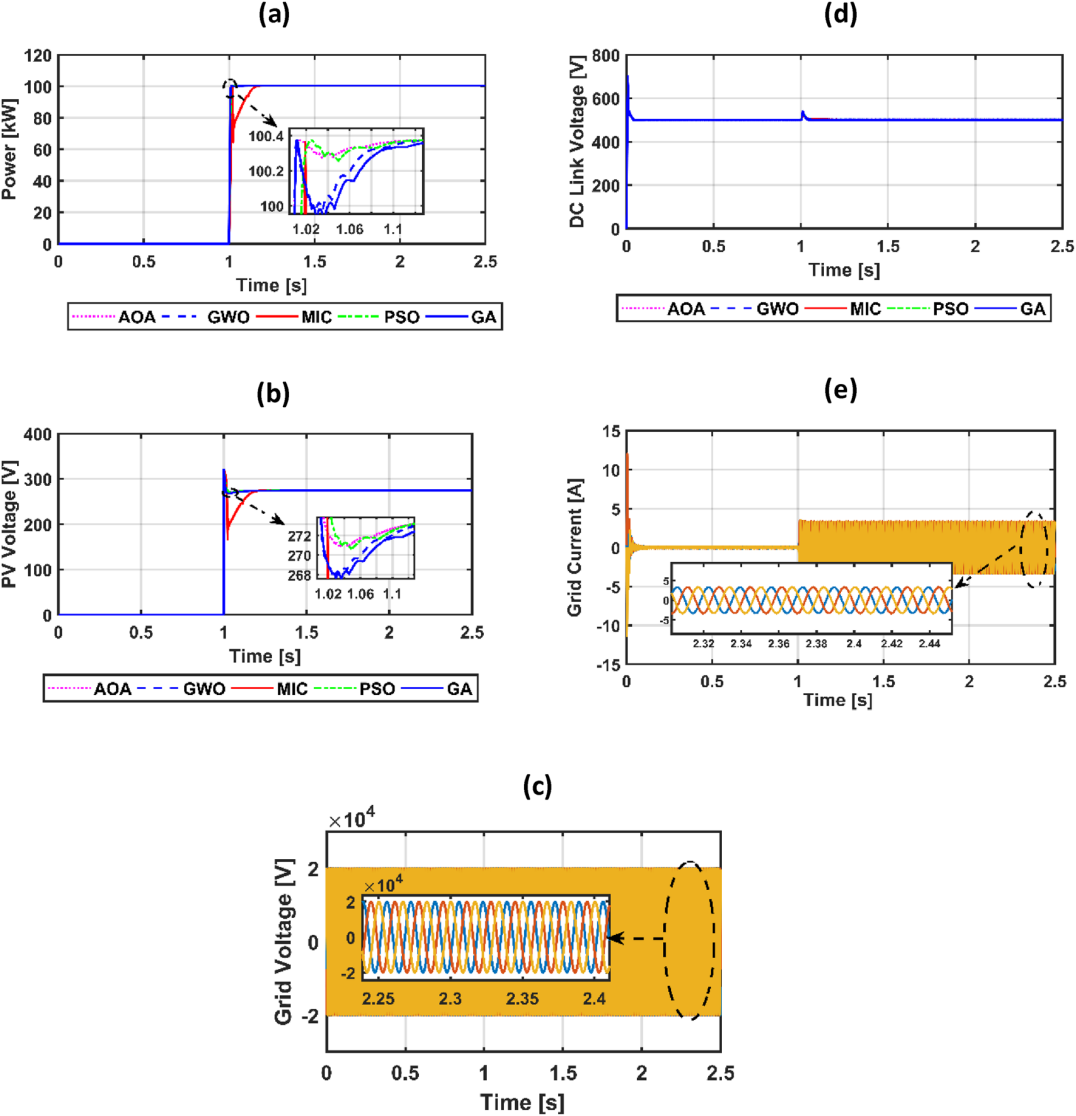
(**a**) PV power, (**b**) PV voltage, (**c**) utility voltage, (**d**) voltage of the DC link, (e) utility current-based AOA.

**Figure 10:**
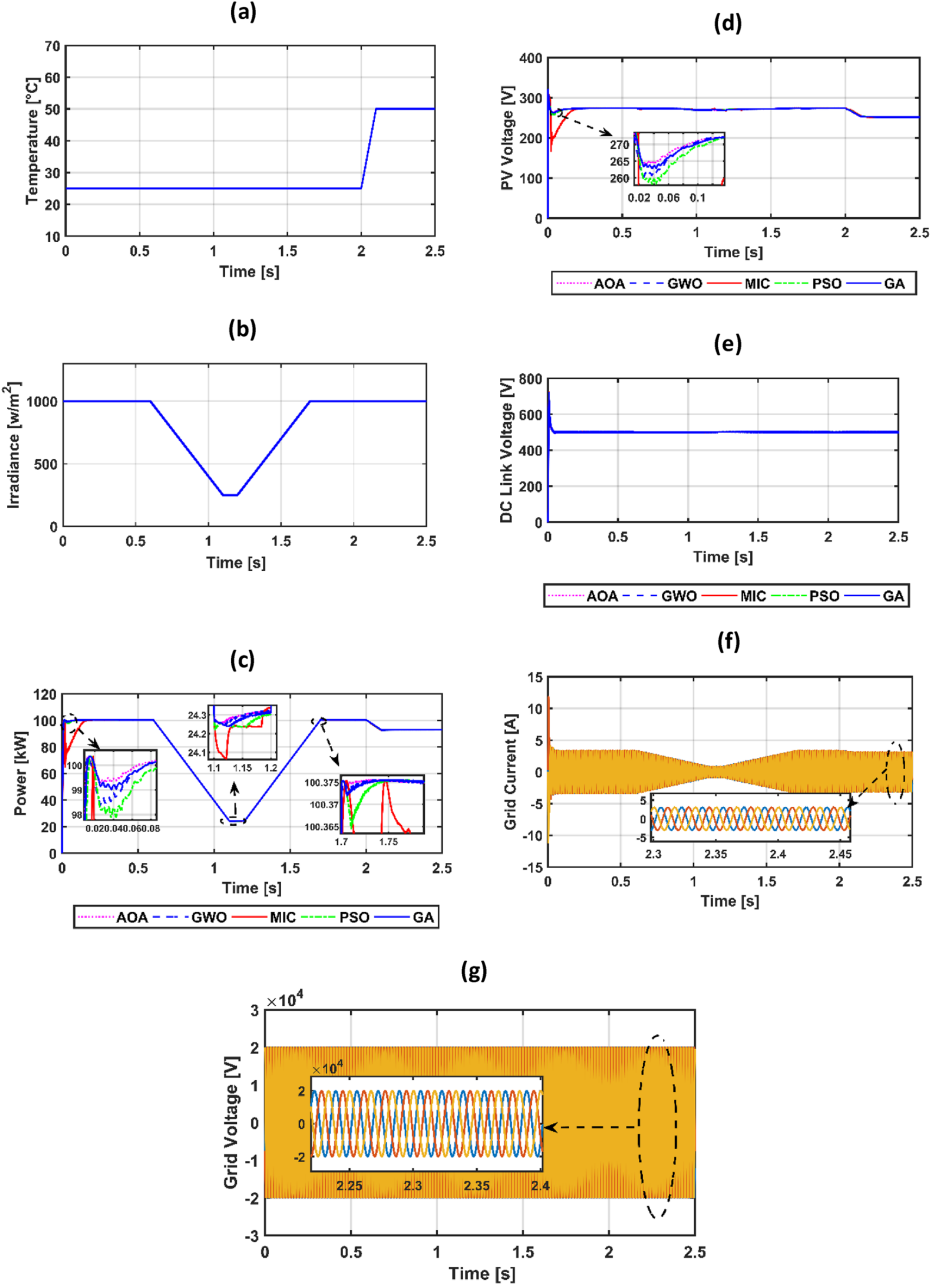
(**a**) ramp temperature, (**b**) ramp irradiance, (**c**) PV power, (**d**) PV voltage, (**e**) voltage of DC link, (**f**) utility current-based AOA, (**g**) utility voltage-based AOA.

**Figure 11:**
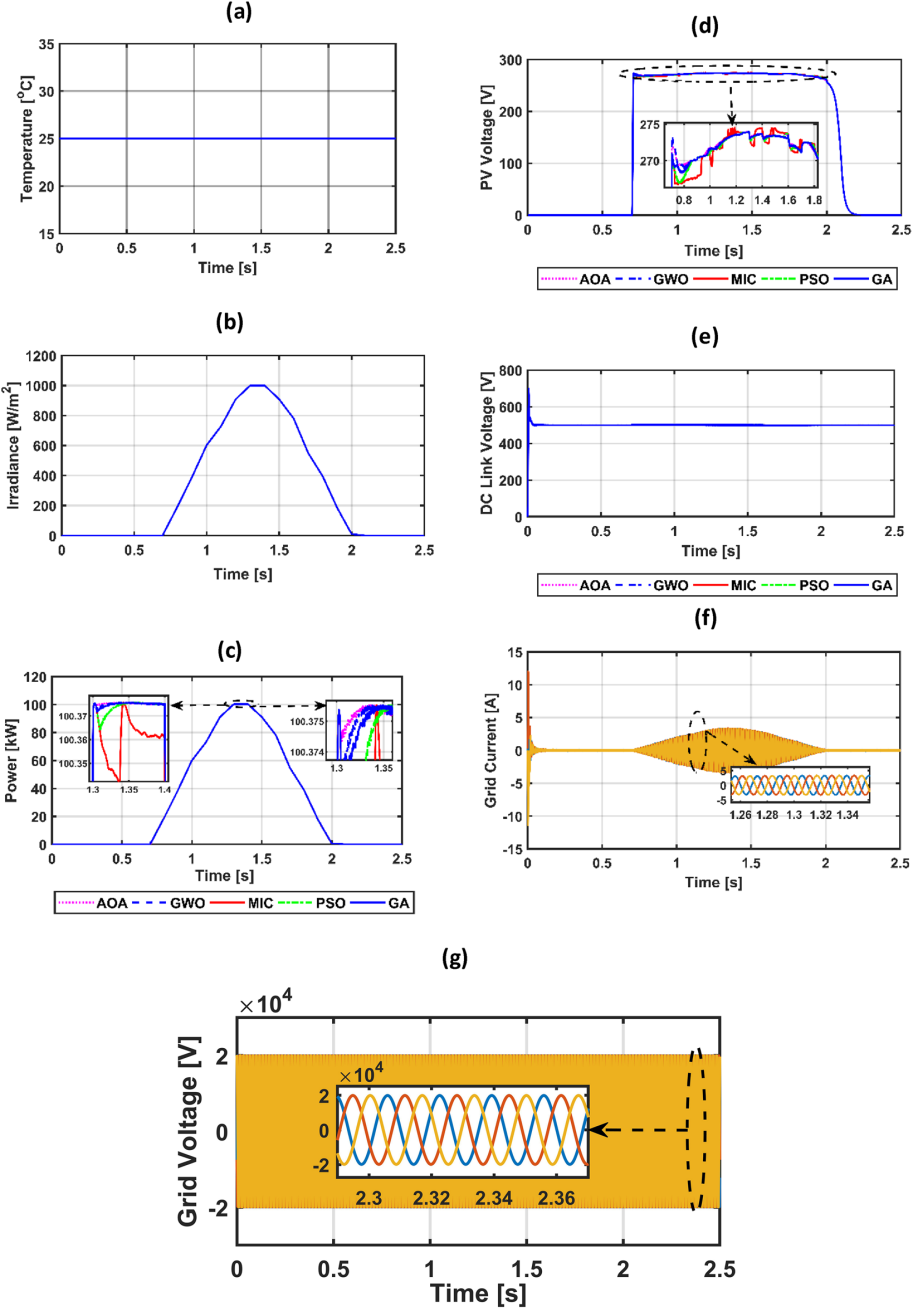
(**a**) fixed temperature, (**b**) different irradiance, (**c**) PV power, (**d**) PV voltage, (**e**) voltage of DC link, (**f**) grid current-based AOA, (**g**) grid voltage-based AOA.

**Figure 12:**
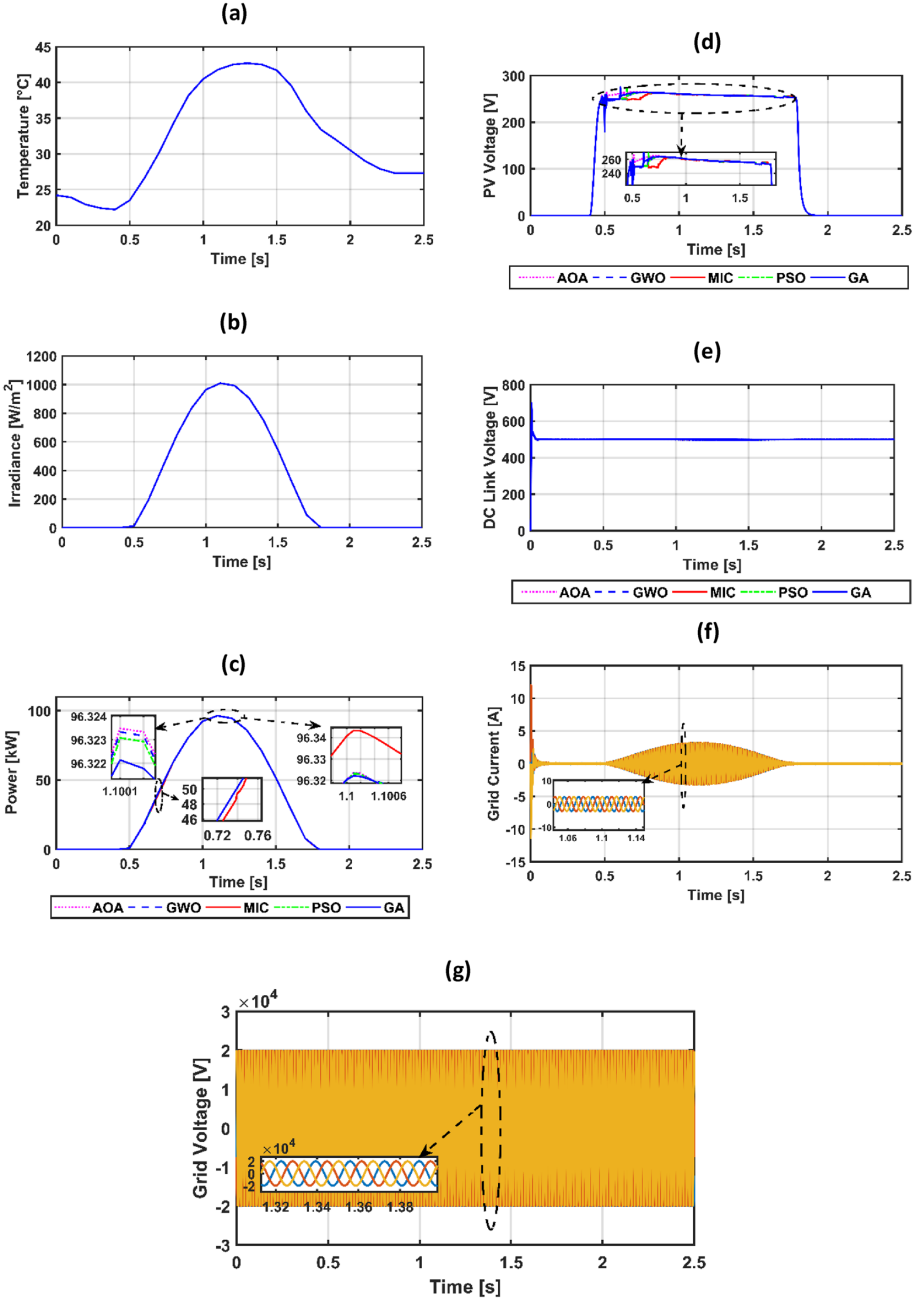
(**a**) realistic temperature, (**b**) realistic irradiance, (**c**) PV power, (**d**) PV voltage, (**e**) voltage of DC link, (**f**) grid current-based AOA, (**g**) grid voltage-based AOA.

**Figure 13:**
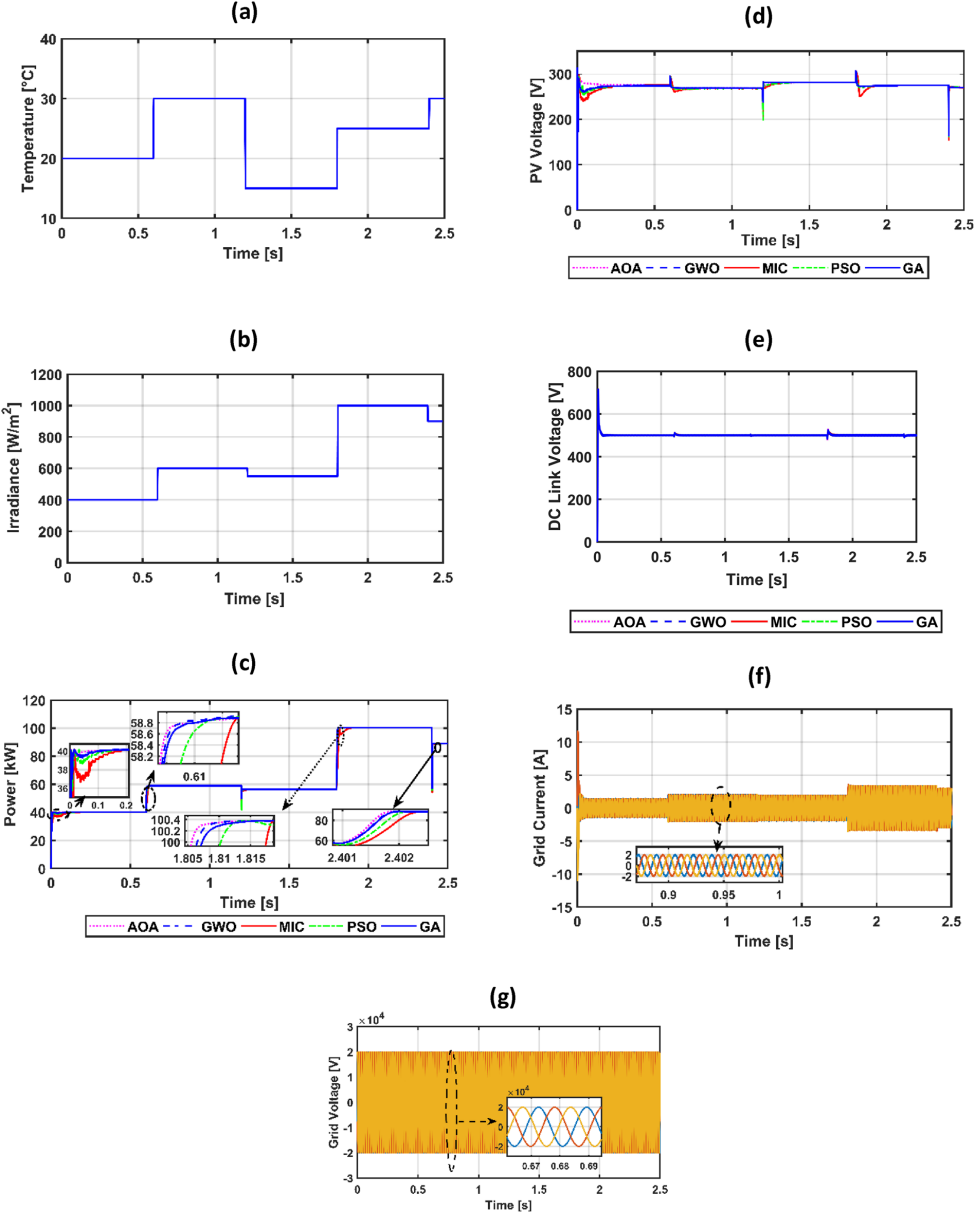
(**a**) variable temperature Level pattern, (**b**) variable Irradiance Level pattern, (**c**) PV power, (**d**) PV voltage, (**e**) voltage of DC link, (**f**) grid current-based AOA, (**g**) grid voltage-based AOA.

The suggested system is evaluated based on five climate weather scenarios. The first scenario is at a constant temperature with a step irradiance pattern shown in Fig. 8a,b. The second is ramp temperature and irradiance shown in Fig. 10a,b. The third one is constant temperature with different irradiance shown in Fig. 11a,b. The fourth one is the realistic temperature and irradiance that were taken at Benban Solar Park, Aswan, Egypt, on May 1, 2019, as shown in Fig. 12a,b. The last one is variable temperature with variable irradiance, as displayed in Fig. 13a,b. The optimum gains of the PI controller obtained from AOA, GWO, GA, and PSO based on four performance indices and (MIC) are pointed out in Table 4. The procedure involves selecting the best of the best performance index of each algorithm, then executing a comparison among AOA, GWO, GA, PSO, and MIC. For the initial case study, the PV power, as well as the PV voltage, have been represented in Fig. 8c,d consecutively based on AOA obtained gains. It is clarified that the best dynamic performance of AOA PV output power occurs with ISE, as it consumes a minimum settling time (0.0103 s) that is scientifically lower than other standards. Furthermore, the minimum power obtained from ISE is 100.275 kW, followed by ITAE at 100.15 kW, IAE at 100.1 kW, and ITSE at 99.9 kW. However, there is no noticeable change in terms of the rise time or overshoot among different standards of error indices. Moreover, the PV output voltage is the smoothest in the case of ISE, as its minimum voltage is 270.8 V, while ITAE is 269.4 V, IAE is 269 V, and ITSE is 267.5 V. Hence, the best standard of AOA is ISE. On the opposite side, Fig. 8e,f presents PV output power and voltage with GWO consecutively. The best one of GWO in PV output response is IAE because it consumes the minimum time (0.067352 s) to settle to the steady-state power, whereas the ITSE that settles at t = 0.079668 s is the second-ranked one, followed by the ISE that settles at t = 0.110601 s and the ITAE that settles at t = 0.1134 s. Moreover, the minimum extracted power by the IAE is 99.96 kW, followed by ISE with 99.94 kW, ITSE with 99.92 kW, and ITAE with 99.8 kW. However, ITSE takes rise time slightly lower than IAE, the IAE settles faster with a slightly lower overshoot. Furthermore, the PV output voltage is the smoothest in the case of IAE over other indices because the minimum voltage attained by IAE is 267.9 V, followed by ISE at 267.8 V, ITSE at 267.65 V, and ITAE at 266.75 V. So, the optimal index of GWO is IAE. Figure 8g,h describes the PV power and voltage-based GA sequentially. It is clarified that the best index of PV power is IAE, as it settles faster compared with the other indices with t = 0.07718 s, followed by ITAE, ISE, and ITSE. In addition, the least achieved power by IAE is 99.92 kW, whereas ITSE is 99.55 kW, ISE is 98.72 kW, and ITAE is 98.6 kW. Additionally, the smoothest PV voltage is obtained by the IAE index, with a minimum voltage of 267.6 V, while ITSE is 265.4 V, ISE is 261.5 V, and ITAE is 261 V. Hence, the best index of GA is IAE. Figure 8i,j defines the PV power and PV voltage-based PSO consecutively. The best PV power curve is obtained by the ITAE, as it is the fastest index to settle at t = 0.0499752 s, followed by the IAE, ITSE, and ISE. Also, the minimum PV power obtained by ITAE is 100.25 kW, whereas IAE is 99.9 kW, ITSE is 99.85 kW, and ISE is 97.6 kW. The PV voltage performance is the best in ITAE with a minimum voltage of 270.6 V, followed by IAE with 267.4 V, ITSE with 267.2 V, and ISE with 257.1 V. Figure 9a,b shows a PV power and voltage based on the best of the best gains of AOA, GWO, MIC, GA, and PSO. The PV power of the PV system equipped with AOA is the best in terms of settling time; the second is PSO; the third best one is GWO; the fourth is GA; but the worst is MIC, as it consumes a longer settling time with large oscillations and has the lowest minimum power of 64.5 kW despite achieving the least overshoot. Besides, the PV voltage in the case of AOA is the smoothest curve, the next is PSO, GWO, GA, and the worst case is MIC. Figure 9c represents the utility three-phase voltage with a phase peak voltage of 20 kV, while Fig. 9d shows the DC link voltage that is set at 500 V and moves to such a reference value except slightly increasing at t = 1 s, which is the instant of step irradiance occurrence from zero to 1000 w/m^2^. Figure 9e provides the AOA grid current that highly affected the irradiance pattern since the temperature is constant. For the second scenario, Fig. 10c,d presents the corresponding dynamic response of PV power and voltage based on AOA, GWO, MIC, GA, and PSO. It is obvious that the power is proportional to the irradiance level and inversely to the temperature. The minimum attained PV power by AOA at t = 0:0.6 s is 99.3 kW, followed by GA at 99 kW, GWO at 98.4 kW, PSO at 97.9 kW, and MIC at 65 kW. Moreover, the AOA is the best response in the low-irradiance case, followed by GWO, GA, PSO, and MIC. Hence, the AOA outperformed GWO, GA, PSO, and MIC whether in steady-state or transient response. Figure 10e–g represents the voltage of the DC link that is a reference set at 500 V, the AOA grid current that follows the irradiance pattern at t = 0:2 s as temperature constant, while decreasing slightly at t = 2:2.1 s as temperature increased, and the AOA grid voltage of peak phase voltage of 20 kV, consecutively. In the third scenario, Fig. 11c provides the dynamic response associated with PV power corresponding with AOA, GWO, GA, PSO, and MIC. As the temperature is not changed, the PV power follows the irradiance level. The three methods succeeded in tracking power over all the irradiance variations. At t = 1.3:1.4 s, the performance of the AOA is the optimal one, while the GWO occupies the second rank, the GA is the third, the PSO is the fourth, and finally, the MIC is the last one. Fig. 11d depicts the PV voltage related to AOA, GWO, GA, PSO, and MIC. PV voltage-based AOA is the smoothest with a minimum voltage of 269.2 V, followed by GWO at 268.95 V, GA at 268.3 V, PSO at 266.9 V, and MIC at 266.7 V. Figure 11e–g provides DC link voltage of 500 V reference value, AOA utility current that follows the irradiance pattern as the temperature constant, and utility voltage with a peak phase voltage of 20 kV, respectively. For the fourth scenario, Fig. 12c presents the PV output power corresponding with AOA, GWO, GA, PSO, and MIC. The MIC power has slightly deviated from AOA, GWO, PSO, and GA from t = 0.65 to 0.78 s. However, the peak power attained by MIC is 96.34 kW, followed by AOA at 96.3235 kW, GWO at 96.3233 kW, PSO at 96.32308 kW, and GA at 96.2214 kW. Figure 12d provides PV voltage. The PV voltage attained by AOA is the fastest one, followed by GA, then GWO, and PSO while MIC takes the longest time to track the voltage. Figure 12e–g represents the DC link voltage of 500 V reference, AOA utility current corresponding to irradiance and temperature variations, and utility voltage, respectively. For the last scenario, Fig. 13c,d presents the corresponding dynamic response of PV power and voltage based on AOA, GWO, GA, PSO, and MIC. All algorithms succeed in tracking the power corresponding to the temperature and irradiation levels. The PV power obtained by AOA is the fastest one to track the power, followed by GWO, GA, PSO, and MIC. The PV voltage is affected inversely by the temperature. Figure 13e–g represents the DC link voltage of 500 V reference, AOA utility current corresponding to irradiance and temperature variations, and utility voltage, respectively.

**Table 4:** Optimal gains of AOA, GWO, MIC, GA, and PSO.

AI Technique	Error criteria	Rise Time(s)	Settling Time (s)	Max. power (kW)	Overshoot Percentage	K_P_	K_I_
AOA	IAE	0.006235	0.0619	100.37553	0.014676	0.0757	1.8024
	ISE	0.006214	0.0103	100.37551	0.01471	0.0807	1.8086
	ITAE	0.006256	0.0603	100.37552	0.01473	0.0909	1.8059
	ITSE	0.006877	0.065	100.37552	0.014675	0.0471	1.7066
GWO	IAE	0.006422	0.067352	100.37553	0.0142213	0.0627	1.729
	ISE	0.00856	0.110601	100.375522	0.014318	0.0866	0.9566
	ITAE	0.008584	0.1134	100.375523	0.01441	0.0711	0.9631
	ITSE	0.006199	0.079668	100.37552	0.0142219	0.067	1.3932
MIC ^49^		0.015982	0.176585	100.37553	0.01419	0.002	0.867
GA	IAE	0.006509	0.07718	100.3755246	0.014524	0.0570	1.3373
	ISE	0.007302	0.098300	100.3755205	0.01446	0.0379	1.1747
	ITAE	0.007133	0.0806242	100.3755266	0.014583	0.0405	1.6368
	ITSE	0.008692	0.110743	100.37552690	0.0146370	0.0596	1.0272
PSO	IAE	0.00620357	0.0772066	100.3755257	0.0145901	0.0667	1.5037
	ISE	0.0080493	0.1080295	100.37552051	0.014320	0.0283	1.0462
	ITAE	0.00850804	0.0499752	100.3755229	0.014552	0.0283	1.0462
	ITSE	0.0085718	0.0975944	1.003755259	0.014668	0.0734	1.1614

## Conclusion

In this article, the AOA technique is utilized for the optimum selection of the parameters of the proposed AOA-based PI-IC-MPPT for a 100-kW grid-connected PV system. The attained result of the suggested control strategy is compared to GWO, MIC, GA, and PSO. Five different scenarios of climate weather conditions are considered, such as constant temperature with step irradiance patterns, ramp irradiance with ramp temperature, the later one is various irradiance with a constant temperature, the fourth one is realistic irradiance and temperature, and the last one is variable irradiance with variable temperature. In the first scenario, the simulation results clarified that AOA reduced the rise time by 61%, 3%, 4.5%, and 26.9% and the settling time by 94%, 84.7%, 86.6%, and 79.3% over MIC, GWO, GA, and PSO in extracting the maximum output of the PV system. Besides, in the second scenario, the dynamic response of AOA outperformed that of GWO, GA, and PSO but MIC is the worst performance even in steady-state or transient response. In the third scenario, all techniques succeed in tracking  the MPP. In the fourth scenario, AOA PV voltage is the fastest, followed by GA, GWO, PSO, and MIC, whereas MIC takes the longest to track the voltage. In the last case study, the PV power obtained by AOA is the fastest one to track the power, followed by GWO, GA, PSO, and MIC. Hence, this study proves the applicability of the new AOA optimization to enhance the dynamic performance of grid-connected PV systems. In future work, the proposed AOA-based IC-MPPT will be hybridized with artificial intelligence techniques for extracting global MPP under various partial shading conditions for grid-connected PV systems.

## Data Availability

The datasets used during the current study are available from the corresponding author upon reasonable request.

## List of symbols

A: Addition arithmetic operator
a: Ideality factor for diode
best (x_j_ ): Position of best-attained solution till now
C_Iter: Current iteration
D: Division arithmetic operator
dI/dV: Incremental conductance term
e: Error
E_g_: Bandgap energy of polycrystalline silicon
G: Solar irradiance
I/V: Instantaneous incremental conductance
I_d_: Diode current
I_o_: Reverse saturation current of the diode
I_ph_: Photogenerated current
I_SCn_: Short circuit current at STC
I_sh_: Current through parallel resistance
K: Boltzmann constant (1.38 × 10^–^^23^ J/K)
K_I_: Integral gain
K_P_: Proportional gain
K_T_: Temperature coefficient
LB_j_: Lower boundary of the ${j}^{\mathrm{th}}$ position
M: Multiplication arithmetic operator
M_Iter: Maximum number of iterations
Max: Maximum values of the accelerated function
Min: Minimum values of the accelerated function
MOA: Math Optimizer Accelerated
MOP: Math Optimizer probability
N_s_: The number of series cells
q: Electron charge (1.6 × 10^–^^19^ C)
r1-r3: Random numbers
R_s_: Series resistance of PV cell
R_sh_: Shunt resistance
S: Subtraction arithmetic operator
T_c_: Temperature of PV cell in Kelvin
t_ss_: Steady-state time response
UB_j_: Upper boundary of the ${j}^{\mathrm{th}}$ position
V: Terminal voltage
V_OCn_: Open circuit voltage at STC
V_t_: Thermal voltage
X_i,j_ (C_Iter + 1): The solution of the next iteration
α: A sensitive parameter
ϵ: A tiny number
μ: Control factor

## Abbreviations

AI: Artificial intelligence
AOA: Arithmetic optimization algorithm
I-V: Current–Voltage relationship
DDM: Double diode model of PV cell
FOSV: Fractional open-circuit voltage
FSCC: Fractional short circuit current
GWO: Grey wolf optimization
IAE: Integral absolute error
IC: Incremental conductance
IRENA: International renewable energy agency
ITAE: Integral time absolute error
ITSE: Integral time square error
MIC: Modified incremental conductance
MPPT: Maximum PowerPoint tracking
P & O: Perturb and observe
PI: Proportional-Integral controller
PV: Photovoltaic
P–V: Power-Voltage relationship
SDM: Single diode model of PV cell
STC: Standard test condition
TDM: Triple diode model
